# Childhood adversity subtypes and violence victimization and perpetration among early adolescents in Shanghai, China

**DOI:** 10.1186/s12887-021-02853-3

**Published:** 2021-09-03

**Authors:** Xiayun Zuo, Ziwei Zhang, Li Yan, Qiguo Lian, Chunyan Yu, Xiaowen Tu, Chaohua Lou

**Affiliations:** 1 NHC Key Lab of Reproduction Regulation (Shanghai Institute for Biomedical and Pharmaceutical Technologies), Fudan University, NO.779 Old Humin Road, Xuhui District, 200237 Shanghai, P.R. China; 2 Wuzhong District Health Committee, Suzhou, Jiangsu P.R. China; 3 Infection Prevention and Control Department, Zhongda Hospital Southeast University, Nanjing, P.R. China

**Keywords:** Adverse childhood experiences (ACEs), Violence, Victimization, Perpetration, Early adolescence

## Abstract

**Background:**

This cross-sectional study aimed to identify adverse childhood experience (ACE) subtypes using variable- and person-centered approaches and examine the possible sex-differentiated associations with violence involvement as victim, perpetrator, and victim-perpetrator.

**Methods:**

Adolescents aged 10–14 years in three junior high schools in Shanghai, China, were selected using a cluster sampling method in November and December 2017. Participants were surveyed anonymously using a computer-assisted self-interview approach via tablets. Thirteen items modified from the CDC-Kaiser ACE study were used to measure the ACEs. Results show subtypes as neglect, abuse, and household dysfunction by developing cumulative index score from the variable perspective and subgroups identified through the latent class analysis (LCA) from the person perspective. Logistic regression analyses were used to test the association between each ACE subtype and violence victimization and perpetration after adjusting for some demographic characteristics.

**Results:**

A total of 1,700 participants were included in the final analysis. Approximately 1,322 (77.76 %) participants reported experiencing at least one ACE. The prevalence of neglect, abuse, and household dysfunction was 64.12 % (*n* = 1090), 61.29 % (*n* = 1042), and 18.24 % (*n* = 310), respectively. Three classes were identified through the LCA: low exposure to all ACEs (*n* = 854, 50.23 %), high exposure to emotional and physical abuse and neglect (n = 715, 42.06 %), and high exposure to all ACEs (*n* = 131,7.71 %). After controlling the covariates, experiencing abuse, neglect, and household dysfunction was significantly related to violence victimization (adjusted odds ratio [aOR] *=* 3.19, 3.29, 2.37, *P* < 0.001) and victim-perpetrator (aOR *=* 3.48, 4.41, 5.16, *P* < 0.001). Adolescent violence perpetration was only found to be associated with being neglected (aOR = 2.37, *P* = 0.003) and suffering household dysfunction (aOR = 3.25, *P* < 0.001). LCA revealed the cumulative effects of ACEs on adolescent violence victimization and perpetration. Sex-stratified analysis indicate that girls were more vulnerable to the negative effects of ACEs, with a higher risk of perpetration among girls exposed to distinctive subtypes or multiple ACEs.

**Conclusions:**

ACEs were ubiquitous and significantly associated with an elevated risk of violence victimization and perpetration during early adolescence. Future research should examine whether these associations persist over time and the intermediating mechanism from the perspectives of individual neurodevelopment, cognition and resilience ability, and social support.

## Background

Early adolescence (10–14 years of age) is a critical turning point in the development of human life, during which adverse childhood experiences (ACEs) can significantly influence the course of life in the short and long terms. ACEs, a set of traumatic stressors experienced before 18 years of age, typically include issues of abuse (physical, emotional, and sexual abuse), neglect (physical and emotional neglect), and household dysfunction (household substance abuse and mental illness, violence between adults and parental incarceration) [1]. Extensive studies on ACEs have suggested that the increase in adverse experiences experienced during childhood is associated with the deterioration of physical and mental health outcomes in adulthood and intergenerational cycles of ACE-related mental health, behavioral, and social problems [2, 3].

Among the many adverse health outcomes, adolescent violence and bullying have become a social issue that has attracted attention due to its high prevalence. Most importantly, the consequences of violence can be severe and persistent, including mental health problems, self-harm, and suicide [4, 5]. Some reports showed that participants who experienced ACEs were more likely to be violent and victims compared to those who did not. For instance, the results of a cross-sectional study from Anhui province in China indicated a significant association between school bullying and child maltreatment, including childhood abuse and neglect, among middle school students [6]. In a birth cohort of 2,232 children in the Environmental Risk Longitudinal Twin Study, child maltreatment and domestic violence were found to be associated with all groups of children involved in bullying (including bullies, victims, and bully-victims) [7]. Myriam and colleagues found linear or curvilinear relationships between cumulative ACEs and types of on-campus violence victimization and perpetration among ninth graders [8].

Despite these findings, many of these studies have focused on specific ACEs, for instance, the specific form of maltreatment (e.g., sexual abuse or physical abuse) or selected aspects of household dysfunction (e.g., witnessing domestic violence), or taking ACEs as a whole, which masked the complexity of these relationships. Disentangling these relationships between different subtypes of ACEs with violence involvement in early adolescence is valuable for the design of interventions using specific components and implementation among the most vulnerable subpopulations. The ACE subtypes were usually generated by discrete groups with similar patterns of ACE exposure [9]. Evidence suggests that in many young people, ACEs with significant interrelations between the different types or groups [10, 11] usually occur simultaneously in multiple rather than simple forms and have cumulative effects [9, 12]. In the surveys undertaken in eight eastern European countries to evaluate ACEs among young adults, the results showed that over half of the respondents reported at least one ACE, and experience of one ACE increased the probability of having other ACEs [13]. Most studies utilized a variable-centered approach to assess the subtypes of multiple childhood adversities based on their nature, such as child sexual abuse, maltreatment, and family dysfunction [6, 14]. In some studies, multiple ACE exposures were measured through the latent class analysis (LCA) from the person-centered perspective to reveal classes of ACEs [9, 15]. The ACE classes often range from low-exposure ACEs to multiple ACEs [9, 12, 15, 16] and the results revealed that young adults exposed to multiple ACEs were at higher risk of alcohol-related problems and psychological symptoms than those who had no or low exposure to ACEs [9, 17]. LCA makes use of the patterning of co-occurrence for different ACEs to classify individuals into unique subtypes that might be etiologically and prognostically distinct [15, 18]. LCA might be a better way to measure the association between ACE typologies and adolescent violence by separating subjects with similar ACE patterns [9].

Diathesis-stress models have proposed both negative life events (i.e., stress) and the individual biological vulnerabilities or cognitions about those events (i.e., diatheses) contributed to the development of internalizing and externalizing psychopathology and impaired social relationships [19, 20]. ACEs, major negative life events during childhood are associated with pervasive health-risk behaviors and social problems including aggressive behaviors, especially throughout childhood and early adolescence [21]. Besides, parental mental health disorders and maltreatment also can be the diatheses of adolescent violence involvement, as either victim or perpetration or both. In this study, data on early adolescents from three junior high schools in Shanghai were used to investigate the association between ACEs and adolescent violence from three aspects: victimization, perpetration, and victim-perpetrator. Two approaches to assess ACE subtypes were utilized—person-centered subtypes by LCA and variable-centered subtypes (abuse, neglect, household dysfunction)—to categorize adolescents according to different patterns of ACE exposure. We hypothesized that (1) there was a high prevalence of ACEs among early adolescents in Shanghai; and (2) ACE subtypes were positively associated with experiences of violence victimization, perpetration, and victim-perpetrator. The evidence surrounding the relationship between ACEs and adolescent violence involvement by sex is inconsistent and needs further exploration [8]. This study also try to explore the pissible sex disparities in the relationship between ACE subtypes and adolescent violence.

## Methods

### Study design and participants

Data were obtained from a survey of the Global Early Adolescent Study (GEAS) in Shanghai, China. GEAS was a multinational study that aimed to understand the development of gender norms among adolescents aged 10–14 years and its impact on adolescent health across time and geography. The survey in Shanghai was administered to three junior high schools in November and December 2017. All adolescents in grades 6, 7, and 8 were selected using the cluster sampling method. Adolescents were surveyed anonymously using a computer-assisted self-interview (CASI) approach via tablets after obtaining informed consent from the participants and their parents or guardians. The electronic questionnaire was set up with logical jumps, reasonable ranges of answers, and other verification procedures. Adolescents were allowed to skip or refuse to answer any of the questions. All procedures were approved by the ethical review boards of the Shanghai Institute of Planned Parenthood Research and the global coordinating institutions (World Health Organization and Johns Hopkins University).

A total of 1,760 records were obtained. The response of “refusal” or “unknown” were coded as missing, except the response of “unknown” regarding registered permanent residence in Shanghai and parental educational level, which could be treated as normal. In variables that contained multiple items, such as ACEs, violence victimization, and perpetration, the variable was considered missing only when all related items were missing; otherwise, the missing value of the item was coded as “no.” Individuals with missing values for variables like parental education level (0.97 %), house renting (1.48 %), parental divorce (1.31 %), local registered permanent residence (0.28 %), and grade (0.22 %) were excluded, resulting in a final sample size of 1,700 (96.59 % of the original sample).

### Measures

#### Adverse childhood experiences (ACEs)

The ACE measurement was modified from the CDC-Kaiser ACE study [1], comprising 13 questions covering 10 ACEs (emotional abuse, physical abuse, sexual abuse, emotional neglect, physical neglect, parental substance abuse, parental emotional distress, mother treated violently, parental incarceration, and household instability). The details of the ACE measurement are shown in Blum’s study [17]. The 10 ACE types could be aggregated into three categories: abuse, neglect, and household dysfunction according to their nature. Every question had three response options: never, sometimes, and often. Dichotomous coding was used, and respondents were defined as exposed to a category or type if they responded “sometimes” or “often” to one or more of the related items.

#### Violence victimization and perpetration

Violence or bullying was measured based on two aspects—victimization and perpetration—each including two typical scenarios that most commonly occurred among adolescents. The questions on victimization were as follows: (a) “have you been teased or called names by someone in the past six months?” and (b) “have you ever been slapped, hit, or otherwise physically hurt by a boy or girl in a way that you did not want in the past six months?” The questions on perpetration were the following: (a) “have you ever bullied or threatened another boy or girl in the past six months?” and (b) “have you ever slapped, hit, or otherwise physically hurt another boy or girl in a way that they did not want in the past six months?” Each question was coded dichotomously (1 = yes, 0 = no). We affirmatively assessed victimization and perpetration if the respondents experienced either of the two incidents. Likewise, victim-perpetrator was assessed affirmatively if the respondents experienced both violence victimization and perpetration.

### Covariates

 Potential covariates, including sex, grade, having siblings or not, parental educational level, renting or owning the house, local registered residence, and parental marital status, were controlled. Most of the covariates were coded dichotomously, except grade (grades 6, 7, and 8) and parental education (junior high school or below, senior high school, college or above, and unknown).

### Statistical analysis

Data were analyzed using Stata version 15.1, and Mplus version 7.4. Pearson chi-square tests were used to assess the prevalence of ACEs and violence involvement between boys and girls. LCA was then performed to explore the person-centered subtypes of ACEs. We investigated the association between the ACE subtypes and violence (victimization, perpetration, and victim-perpetrator) from variable-centered and person-centered perspectives using logistic regression models, while controlling for covariates. Sex was not included in the sex-stratified logistic regression model. *P <* 0.05 was considered statistically significant, for two-sided tests.

### Latent class analysis

Person-centered ACE subtypes were identified by LCA, including 10 ACE indicators, and six models were estimated using Mplus version 7.4. We reported the following model fit statistics: log likelihood, Akaike information criteria, Bayesian information criterion (BIC), adjusted BIC, entropy, and bootstrapped likelihood ratio test, as shown in Table 1. A three-class model of ACEs was identified as the best model based on joint consideration of the full range of model fit statistics, the theoretical significance of the ACE classes, and the interpretation of the clusters and class size [12].

**Table 1:** Fit indices for the latent class models with one to six classes of ACEs

Class number	*LL* (model)	AIC	BIC	Adjusted BIC	Entropy	BLRT *P-*value
Class 1	720.910	13104.66	13158.550	13126.781	NA	NA
Class 2	549.713	12055.835	12170.041	12103.327	0.68	0.0000
Class 3	382.637	11880.998	12055.026	11953.366	0.71	0.0000
Class 4	351.958	11867.730	12101.580	11964.975	0.62	0.0200
Class 5	323.188	11863.442	12157.115	11985.563	0.63	0.1538
Class 6	307.129	11856.514	12210.009	12003.512	0.67	0.0400

Abbreviations: *LL* log likelihood; *AIC* Akaike information criteria; *BIC* Bayesian information criterion; *BLRT* bootstrapped likelihood ratio test; *NA* not applicable

## Results

### Sample characteristics

A total of 1,700 participants were included in the final analysis, including 865 boys (50.88 %) and 835 girls (49.12 %). There were 579 (34.06 %), 633 (37.24 %), and 488 (28.71 %) subjects aged 10–14 years (*M* = 12.46, *SD* = 0.96) in grades 6, 7, and 8, respectively. Moreover, 1,098 subjects (64.59 %) were from only-child families, 1,348 (79.29 %) had local registered permanent residences, and 312 (18.35 %) lived in apartments. Regarding the highest parental education level, the reported proportion of participants whose parents’ education level were junior high school and below, high school or technical school, college or university graduates, and unknown were 10.76 % (183), 26.53 % (451), 50.41 % (857), and 12.29 % (209), respectively. There were 195 (11.47 %) subjects whose parents were divorced. No statistical differences in these demographic characteristics were observed, except age between boys and girls (see Table 2).

**Table 2:** Demographic characteristics of the sample (%)

Variables	Total	Boys	Girls	*P*
	*N* = 1700	*N* = 865	*N* = 835	
**Age (M [SD])Grade**	12.46[0.96]	12.51[0.98]	12.40[0.93]	0.013
6	34.06	33.41	34.73	
7	37.24	36.42	38.08	0.395
8	28.71	30.17	27.19	
**Has siblings**				
Yes	35.41	36.98	33.89	0.198
No	64.59	63.12	64.59	
**Locally registered resident**				
Yes	79.29	77.80	80.84	0.298
No	20.71	22.20	19.16	
**Rents a house**				
Yes	18.35	20.12	16.53	0.056
No	81.65	79.88	83.47	
**Highest parental education**				
Junior high school and below	10.76	12.49	8.98	
High/technical school	26.53	25.78	27.31	
College/university	50.41	49.13	51.74	0.115
Unknown	12.29	12.60	11.98	
**Parental divorce**				
Yes	11.47	10.06	12.93	0.063
No	88.53	89.94	88.53	

Abbreviation: *M [SD]* mean [standard deviation]

### Prevalence of ACE subtypes and violence involvement

The prevalence of overall and each subtype of ACEs is shown in Table 3. About 77.76 % of the participants reported experiencing at least one ACE. Moreover, 64.12 and 61.29 % of the participants had experienced neglect and abuse, respectively. The incidence of household dysfunction was relatively low (18.24 %). Emotional abuse (56.94 %) and emotional neglect (51.76 %) were the most commonly reported types of abuse and neglect. More than one-third of participants reported experiencing physical abuse (38.15 %) and physical neglect (38.12 %). In terms of household dysfunction, the most prevalent ACE was parental emotional distress (8.32 %), and parental incarceration (2.36 %) was the least reported. Generally, the self-reported rates of physical neglect (43.98 % for boys vs. 32.15 % for girls, *P* < 0.001), sexual abuse (9.94 % for boys vs. 6.35 % for girls, *P* = 0.007), and parental substance abuse (5.01 % for boys vs. 2.11 % for girls, *P* = 0.002) were higher among boys than in girls, while the opposite was true for emotional neglect (49.02 % for boys vs. 54.61 % for girls, *P* = 0.021).

**Table 3:** Prevalence of ACE subtypes and adolescent violence (%)

	Total	Boys	Girls	*P*
	*N* = 1700	*N* = 865	*N* = 835	
**Any category reported**	77.76	79.08	76.41	0.186
**Variable-centered subtypes**				
**Abuse**	61.29	63.01	59.52	0.140
Emotional abuse	56.94	56.26	57.64	0.579
Physical abuse	38.15	39.21	37.06	0.375
Sexual abuse	8.18	9.94	6.35	0.007
**Neglect**	64.12	64.39	63.83	0.810
Emotional neglect	51.76	49.02	54.61	0.021
Physical neglect	38.12	43.98	32.15	< 0.001
**Household dysfunction**	18.24	19.65	16.77	0.123
Parental substance abuse	3.57	5.01	2.11	0.002
Parental emotional distress	8.32	8.25	7.38	0.172
Mother treated violently	5.19	4.61	5.79	0.281
Parental incarceration	2.36	1.91	2.82	0.225
Household instability	7.18	7.05	7.31	0.840
**Person-centered subtypes**				
Class 1 – low ACEs	50.23	49.94	50.54	0.820
Class2 – high neglect and abuse	42.06	42.66	41.44	
Class 3 – high ACEs	7.71	8.02	7.40	
**School bullying**				
Victimization	36.59	42.43	30.54	< 0.001
Perpetration	7.06	8.79	5.27	0.005
Victim-perpetrator	5.82	7.51	4.07	0.002

Abbreviation: *ACE* adverse childhood experience

The LCA identified three classes of ACEs. Class 1 was characterized by low exposure to all ACEs (48.94 %); Class 2 with high exposure to emotional and physical abuse and neglect (42.06 %), that is, moderate exposure; and Class 3 with a high exposure to all ACEs (9.00 %). Participants in Class 1 had a low probability of experiencing any ACEs (< 0.25), while those in Class 2 were characterized by a high probability of emotional neglect (0.77) and emotional abuse (0.95), as well as physical neglect (0.45) and physical abuse (0.66). Furthermore, participants in Class 3 were distinguished by higher levels of all ranges of ACE exposure (0.30–0.79 for abuse, 0.70–0.95 for neglect, 0.18–0.46 for household dysfunction). Sex differences were not obvious in the distribution of subjects to the three classes (Table 3).

It was found that 36.59 % of adolescents had been victims of violence or bullying, 7.06 % were perpetrators, and 5.82 % were involved in victimization and perpetration in the past six months. Sex differences were apparent in the prevalence of victimization (42.43 % for boys vs. 30.54 % for girls, *P* < 0.001), perpetration (8.79 % for boys vs. 5.27 % for girls, *P* = 0.005), and victim-perpetrator (7.51 % for boys vs. 4.07 % for girls, *P* = 0.002).

### Relationships between ACE subtypes and violence involvement

Chi-square analysis showed that any ACEs reported or any ACE subtype reported was associated with violence victimization, perpetration, and victim-perpetrator. Young adolescents who reported any ACE; experienced abuse, neglect, and family dysfunction; or were exposed to moderate (Class 2) and high levels (Class 3) of ACEs had higher percentages of victimization, perpetration, and victim-perpetrator (Table 4).

**Table 4:** Chi-square analysis on the bivariate relationships between ACE subtypes and adolescent violence

	Perpetration			Victimization			Victim-perpetrator		
	Overall	Boys	Girls	Overall	Boys	Girls	Overall	Boys	Girls
	*N* = 1700	*N* = 865	*N* = 835	*N* = 1700	*N* = 865	*N* = 835	*N* = 1700	*N* = 865	*N* = 835
**Any ACE reported**									
0	1.85	3.31	0.51	14.29	17.13	11.68	1.32	2.21	0.51
1	8.55	10.23	6.74	42.97	49.12	36.36	7.11	8.92	5.17
* P*	< 0.001	0.004	0.001	< 0.001	< 0.001	< 0.001	< 0.001	0.002	0.004
**Variable-centered subtypes**									
Abuse									
0	3.50	5.63	1.48	20.36	22.81	18.05	2.28	3.75	0.89
1	9.31	10.64	7.85	46.83	53.94	39.03	8.06	9.72	6.24
* P*	< 0.001	0.012	< 0.001	< 0.001	< 0.001	< 0.001	< 0.001	0.001	< 0.001
Neglect									
0	2.46	3.90	0.99	19.18	25.65	12.58	1.80	3.25	0.33
1	9.63	11.49	7.69	46.33	51.71	40.71	8.07	9.87	6.19
* P*	< 0.001	< 0.001	< 0.001	< 0.001	< 0.001	< 0.001	< 0.001	< 0.001	< 0.001
Household dysfunction									
0	4.39	5.61	3.17	31.73	36.26	27.19	3.38	4.32	2.45
1	19.03	21.76	15.71	58.39	67.65	47.14	16.77	20.59	12.14
* P*	< 0.001	< 0.001	< 0.001	< 0.001	< 0.001	< 0.001	< 0.001	< 0.001	< 0.001
**Person-centered subtypes**									
Class 1	3.16	5.32	0.80	21.31	25.93	16.59	2.22	3.94	0.47
Class 2	9.09	10.30	7.80	48.95	56.10	41.33	7.69	9.21	6.07
Class 3	21.37	23.44	19.40	68.70	75.00	62.69	19.08	21.88	16.42
* P*	< 0.001	< 0.001	< 0.001	< 0.001	< 0.001	< 0.001	< 0.001	< 0.001	< 0.001

Abbreviation: *ACE* adverse childhood experience

Considering a statistically significant correlation between violence victimization and perpetration (r = 0.26; *P* < 0.001), in addition to controlling demographic variables, victimization and perpetration were mutually controlled in the multivariate regression analyses. Table 4 shows the results of the multivariate logistic regression analysis for violence victimization with covariates controlled, demonstrating a positive association between any ACE reported and violence victimization (adjusted odds ratio [aOR], 3.99; 95 % confidence interval [CI], 2.91, 5.48). When the variable-centered subtype of ACEs was recorded as a dichotomous variable separately, respondents’ experiences of abuse, neglect, and household dysfunction in childhood were significantly correlated with violence victimization (aOR *=* 3.19, 3.29, 2.37, respectively; *P* < 0.001). Compared to the respondents in Class 1, those in Class 2 (aOR, 3.36; 95 % CI, 2.67, 4.22) and Class 3 (aOR, 6.93; 95 %CI, 4.49, 10.70) were more likely to be victims, which exhibited a dose-response effect of ACEs on adolescent violence victimization. Multivariate analysis stratified by sex showed similar results among male and female adolescents (Table 5).

**Table 5:** Adjusted odds ratios for adolescent violence victimization according to ACE subtypes

	Overall		Boys		Girls	
	aOR (95 % CI)	*P*	aOR (95 % CI)	*P*	aOR (95 % CI)	*P*
Any ACE reported	3.99 (2.91, 5.48)	< 0.001	4.43 (2.88, 6.82)	< 0.001	3.50 (2.18, 5.62)	< 0.001
**Variable-centered subtypes**						
Abuse	3.19 (2.52, 4.04)	< 0.001	4.02 (2.80, 5.57)	< 0.001	2.46 (1.75, 3.48)	< 0.001
Neglect	3.29 (2.56, 4.19)	< 0.001	2.83 (2.06, 3.89)	< 0.001	4.06 (2.75, 6.01)	< 0.001
Household dysfunction	2.37 (1.80, 3.12)	< 0.001	3.07 (2.10, 4.50)	< 0.001	1.70 (1.12, 2.57)	0.012
**Person-centered subtypes**						
Class 1						
Class 2	3.36 (2.67, 4.22)	< 0.001	3.66 (2.68, 5.00)	< 0.001	3.07 (2.17, 4.35)	< 0.001
Class 3	6.93 (4.49, 10.70)	< 0.001	7.66 (4.03, 14.55)	< 0.001	6.40 (3.50, 11.69)	< 0.001

Abbreviations: *ACE* adverse childhood experience; *aOR* adjusted odds ratio, *CI* confidence interval

As shown in Table 6, after controlling for the effects of covariates, any ACE reported was associated with adolescent violence perpetration (aOR, 2.44; 95 %CI, 1.09, 5.43). Two ACE subtypes, neglect and household dysfunction, were significantly related to violence perpetration (aORs were 2.37 and 3.25, respectively). Among subjects in Classes 2 and 3 who reported moderate and high exposure to ACEs, the risk of perpetration was higher compared to those in Class 1, with aOR *=* 1.79 (95 % CI: 1.09, 2.91) and aOR *=* 3.45 (95 % CI: 1.83, 6.51), respectively. Sex-stratified analysis implied that girls were more vulnerable to the negative effects of ACEs. Girls who experienced abuse or neglect were more likely to be perpetrators, but this was not the case among boys. Analysis from the person-centered analysis also demonstrated that the aORs of girls who were likely to be perpetrators with moderate and high ACE exposures were 5.70 and 11.95, respectively, while no significant correlations were found among boys

**Table 6:** aORs for adolescent violence perpetration according to ACE subtypes

	Overall		Boys		Girls	
	aOR (95 % CI)	*P*	aOR (95 % CI)	*P*	aOR (95 % CI)	*P*
Any ACE reported	2.44 (1.09, 5.43)	0.029	1.54 (0.62, 3.79)	0.349	6.97 (0.93, 52.53)	0.059
**Variable-centered subtypes**						
Abuse	1.53 (0.93, 2.51)	0.090	0.95 (0.52, 1.73)	0.865	3.54 (1.34, 19.40)	0.011
Neglect	2.37 (1.34, 4.22)	0.003	1.90 (0.98, 3.68)	0.059	4.29 (1.26, 14.62)	0.020
Household dysfunction	3.25 (2.12, 4.96)	< 0.001	2.76 (1.62, 4.69)	< 0.001	4.44 (2.14, 9.23)	< 0.001
**Person-centered subtypes**						
Class 1						
Class 2	1.79 (1.09, 2.91)	0.020	1.03 (0.57, 1.85)	0.927	5.70 (1.91, 17.06)	0.002
Class 3	3.45 (1.83, 6.51)	< 0.001	2.07 (0.93, 4.62)	0.074	11.95 (3.35, 42.59)	< 0.001

Abbreviations: *ACE* adverse childhood experience; *aOR* adjusted odds ratio, *CI* confidence interval

A positive association was observed between being victim-perpetrator in violence and any ACE reported after adjusting for covariates (aOR, 5.09; 95 % CI, 2.04, 12.68). It was also observed that abuse, neglect, and household dysfunction were associated with a high risk of being victim-perpetrator (aOR *=* 3.48, 4.41, 5.16, respectively; *P* < 0.001). LCA also showed a higher probability of being the victim-perpetrator in groups suffering from emotional and physical maltreatment (Class 2: aOR *=* 3.49, 95 % CI, 2.04, 5.98) and groups with high ACEs (Class 3: aOR *=* 8.99, 95 % CI, 4.66, 17.37). Sex stratification analysis also showed significant associations both among boys and girls. Compared with boys, girls in any ACE subtype had a greater likelihood of being victim-perpetrator, either from variable-centered or person-centered perspectives (Table 7).

**Table 7:** aORs for adolescent being victim-perpetrator in violence according to ACEs subtypes

	Overall		Boys		Girls	
	aOR (95 % CI)	*P*	aOR (95 % CI)	*P*	aOR (95 % CI)	*P*
Any ACE reported	5.09 (2.04, 12.68)	< 0.001	3.79 (1.35, 10.64)	0.012	9.45 (1.27, 70.30)	0.028
**Variable-centered subtypes**						
Abuse	3.48 (1.98, 6.12)	< 0.001	2.51 (1.31, 4.81)	0.006	6.90 (2.07, 23.01)	0.002
Neglect	4.41 (2.33, 8.37)	< 0.001	3.00 (1.50, 6.01)	0.002	18.38 (2.48, 136.06)	0.004
Household dysfunction	5.16 (3.33, 7.99)	< 0.001	5.19 (3.03, 9.02)	< 0.001	5.09 (2.35, 11.04)	< 0.001
**Person-centered subtypes**						
Class 1 (low)						
Class 2 (moderate)	3.49 (2.04, 5.98)	< 0.001	2.27 (1.24, 4.17)	0.008	13.32 (3.07, 57.73)	0.001
Class 3 (high)	8.99 (4.66, 17.37)	< 0.001	5.88 (2.65, 13.02)	< 0.001	35.08 (7.15, 172.01)	< 0.001

Abbreviations: *ACE* adverse childhood experience; *aOR* adjusted odds ratio, *CI* confidence interval

## Discussion

In this study, we explored the prevalence of ACE subtypes and their association with violence from the perspectives of victimization, perpetration, and victim-perpetrator using a sample of early adolescents in three junior high schools in Shanghai, China. The present study showed a high prevalence of ACE exposure and significant associations between subtypes of childhood adversities and violence, either victimization or perpetration.

It was observed that over three quarters of the respondents had experienced at least one type of ACEs. The reporting rate of ACEs was similar to the results of a cohort study of an urban minority sample in the United States [22]. However, the reporting rate was higher than that in other studies. Surveys in eight eastern European countries with 10,696 respondents aged 18–25 years showed that over half of the respondents (53.6 %) reported at least one ACE [13]. Among the ACE subtypes, emotional abuse (56.94 %) and emotional neglect (51.76 %) were the most commonly reported, followed by physical abuse (38.15 %) and physical neglect (38.12 %), suggesting a high prevalence of maltreatment and staggering incidence of emotional maltreatment. In a cross-sectional survey of 520 respondents from regional primary healthcare centers in Bosnia and Herzegovina, it was found that emotional neglect was the most common type of ACE (25.6 %) and was significantly more prevalent among women [23]. Another study showed that at least 30 % of participants reported emotional neglect and physical neglect, while 40 % reported emotional abuse [24]. A possible explanation for the higher prevalence of maltreatment, especially emotional abuse and neglect, found in our study could be the prevalent Chinese culture that regards harsh parental discipline as an embodiment of parental involvement, guidance, and love. As one of the most prevalent disciplinary techniques in Chinese families, harsh parental discipline involves parenting with higher levels of power assertion, behavioral and psychological control, psychological aggression, and physical abuse [25, 26].

ACEs tended to occur in clusters, such that people who reported experiencing at least one ACE were likely to have experienced multiple ACEs [2]. Therefore, we utilized two approaches to assess ACE subtypes and estimate the correlation between adverse subtypes and violence involvement. One approach showed nearly all significant associations between variable-centered ACE subtypes (abuse, neglect, household dysfunction) and forms of violence, with the exception of the associations of abuse and neglect on boys’ violence perpetration. The person-centered approach, LCA, suggested graded relationships between the different co-occurring ACE patterns and the probability of violence victimization and perpetration. The two approaches corroborated each other in disclosing the distinct association of the subtypes, that is, neglect, abuse, and family dysfunction, as well as the cumulative effects of ACEs. Our findings were also partially corroborated by other studies on early and late adolescents. In a study of 34 high school students in grades 10, 11, and 12, both sexually abused girls and boys were found to be more likely to experience bullying than teenagers without a history of child sexual abuse [27]. The Minnesota Student Survey, with a large sample of students in grades 6, 9, and 12, found that the likelihood of adolescent violence-related perpetration and victimization increased as the number of adverse events identified by the youth increased. ACEs should be considered as risk factors for a spectrum of violence-related outcomes during adolescence [8, 14].

There may be several reasons for the correlation between ACEs and violence involvement that emerge even during early adolescence, as revealed by this study. First, it has been shown previously that ACEs tend to cluster within populations [28]. In other words, other types of ACEs will likely arise in a child with ACEs [29], accounting for this cumulative effect. Second, adolescents who suffer from more types or more severe ACEs tend to experience more psychological distress, feel more powerless, and have lower self-esteem, making them more vulnerable to being bullied. Moreover, the developmental victimology framework showed that many kinds of victimization had common risk factors such as family instability and the lack of supervision [30], which provided theoretical support for our research results. Third, social learning theories assume that adolescents copy violent behaviors based on their experiences or observation from others [31, 32]. Children’s family experiences and parenting behaviors help shape their capacity to adapt and cope at school and have an impact on children’s peer relationships [33]. Conversely, trauma and stress theories assert that continued experiences such as maltreatment (trauma) or persistent psychological distress associated with material hardship (stress) put an individual at increased risk of later perpetration [34, 35]. These findings also explain why teenagers who suffer from ACEs are more likely to bully others.

This study found notable sex differences in the relationship between ACEs and violence perpetration. Compared with boys, girls were seemingly more vulnerable to violence perpetrators when experiencing ACEs reflected either by variable-centered or person-centered analysis. This may be due to sex-differentiated sensitivity to the environment and the potential bidirectional effects between social and cognitive systems in adolescent years [15]. Girls in early adolescence were more vulnerable and sensitive than boys, who, through exposed adversities, develop attachment difficulties, including poor emotional regulation, the lack of trust, and the fear of getting close to others [36], making them more vulnerable to violence. The sex-differentiated association between the ACE subtypes and adolescent violence involvement implies that greater attention should be paid to the intermediating factors or mechanisms from the perspectives of individual neurodevelopment, cognition and resilience ability, and social support through the path between ACEs and adolescent violence.

Although the relationship between ACEs and adolescent violence is undoubtedly complex and influenced by other factors, some implications can still be considered. Preventing exposure to ACEs in early adolescence may be crucial in preventing further violence involvement. Preventive interventions designed to reduce ACEs and adolescent violence are equally important in promoting and maintaining the physical and mental health of adolescents [9]. The context in Shanghai showing a high prevalence of emotional and physical abuse and neglect and their associations with adolescent violence pointed out the importance of criticizing the Chinese traditional parental discipline. It also implies an important policy strategy for improving the connections between family and school environments, while focusing on ACEs and school bullying, as a key mediator for the subsequent development and maintenance of adolescent physical and mental health [15].

Our study had several limitations. First, the study had a cross-sectional design, which hindered its ability to infer causality. The correlation between the ACE subtypes and adolescent violence may be bidirectional, and further longitudinal studies are needed. Second, the self-reported experience of ACEs, victimization, and perpetration may result in underestimation due to social discrimination. This survey was conducted using CASI technology, which could improve the privacy of children and reduce information bias. Third, the sample came from junior high schools in urban Shanghai considering that adolescents aged 10–14 years were all enrolled in school with the national policy of 9-year mandatory education. However, our findings cannot be generalized to other groups of adolescents in China.

## Conclusions

Despite the limitations, our findings suggest that ACEs are common and could elevate the risk of violence involvement as victim, perpetrator, and victim-perpetrator during early adolescence. Girls experiencing multiple ACEs were more vulnerable to violence perpetration. Future research should examine whether these associations persist over time and sex-differentiated intermediating mechanisms from the perspectives of individual neurodevelopment, cognition and resilience ability, and social support.

## Data Availability

The datasets used and/or analysed during the current study are available from the corresponding author on reasonable request.

## Abbreviations

ACEs: Adverse Childhood Experiences
LCA: Latent class analysis
GEAS: Global Early Adolescent Study
CDC: Centers for Disease Control and Prevention
CASI: Computer-Assisted Self-Interview
LL: Log-likelihood
AIC: Akaike information criteria
BIC: Bayesian information criterion
BLRT: Bootstrapped Likelihood-Ratio Test
aOR: Adjusted odds ratio
CI: Confidence interval
